# Comparative Cardiovascular Outcomes of SGLT2i Plus Low-Dose of Conventional Triple Therapy Versus High-Dose of Conventional Triple Therapy for Heart Failure with Reduced Ejection Fraction (HFrEF): A Retrospective Cohort Study

**DOI:** 10.3390/medicina62040781

**Published:** 2026-04-17

**Authors:** Suwat Khamboonruang, Parita Bunditboondee, Pongpun Jittham, Surarong Chinwong

**Affiliations:** 1 Master’s Degree Program in Clinical Pharmacy, Faculty of Pharmacy, Chiang Mai University, Chiang Mai 50200, Thailand; wajn@live.com; 2 Department of Pharmacy, Chiangrai Prachanukroh Hospital, Chiang Rai 57000, Thailand; 3 Division of Cardiovascular Medicine, Department of Medicine, Ratchaburi Hospital, Ratchaburi 70000, Thailand; meaw_pao@hotmail.com; 4 Division of Cardiovascular Medicine, Department of Medicine, Faculty of Medicine, Naresuan University, Phitsanulok 65000, Thailand; boy_pong@yahoo.com; 5 Cardiac Center, Naresuan University Hospital, Phitsanulok 65000, Thailand; 6 Department of Pharmaceutical Care, Faculty of Pharmacy, Chiang Mai University, Chiang Mai 50200, Thailand; 7 Research Center for Innovation in Analytical Science and Technology for Biodiversity-Based Economic and Society (I-ANALY-S-T_B.BES-CMU), Multidisciplinary Research Institute (MDRI), Chiang Mai University, Chiang Mai 50200, Thailand

**Keywords:** heart failure, SGLT2 inhibitors, guideline-directed medical therapy, dosage

## Abstract

*Background and Objectives*: Sodium-glucose cotransporter 2 inhibitors (SGLT2i) reduce cardiovascular (CV) death and heart failure hospitalizations (HFH) in patients with heart failure with reduced ejection fraction (HFrEF). However, data regarding their use in combination with different doses of guideline-directed medical therapy (GDMT) remain limited. This study aimed to evaluate whether SGLT2i combined with low-dose conventional triple therapy is non-inferior to high-dose conventional triple therapy in preventing adverse cardiovascular outcomes. *Materials and Methods*: This retrospective observational study included 334 patients with HFrEF treated between 31 March 2018 and 31 March 2024. Of these, 110 received SGLT2i plus low-dose conventional triple therapy, and 224 received high-dose conventional triple therapy. A non-inferiority framework was applied to compare outcomes between groups. The primary endpoint was a composite of CV death and HFH, while secondary endpoints included the individual components. *Results*: The composite endpoint occurred more frequently in the SGLT2i plus low-dose group. After inverse probability of treatment weighting and multivariable Cox analysis, this group demonstrated a significantly higher risk of the composite outcome (adjusted HR 4.10, 95% CI 2.07–8.13; *p* < 0.001). CV death was similar between groups; however, HFH was significantly more frequent in the SGLT2i plus low-dose group. *Conclusions*: In patients with HFrEF, SGLT2i combined with low-dose conventional triple therapy did not demonstrate comparable clinical outcomes to high-dose conventional triple therapy in reducing CV death and HFH, particularly in patients with a higher baseline burden of disease severity. These findings underscore the importance of optimizing background GDMT dosing alongside the incorporation of SGLT2i into clinical practice.

## 1. Introduction

Heart failure with reduced ejection fraction (HFrEF) is characterized by a reduced left ventricular ejection fraction (LVEF) ≤ 40%. Episodes of decompensation require hospitalization and are associated with increased mortality and reduced quality of life. Current treatment guidelines recommend that all patients with HFrEF receive guideline-directed medical therapy (GDMT), comprising four key drug classes [1,2]. Titration to the maximally tolerated dose is strongly recommended to optimize therapeutic outcomes. The first class is renin–angiotensin system inhibitors (RASi), which encompass angiotensin-converting enzyme inhibitors (ACEi), angiotensin receptor blockers (ARB), and angiotensin receptor–neprilysin inhibitors (ARNi), all of which act on the renin–angiotensin–aldosterone system. The second class comprises beta-blockers (BB), which attenuate sympathetic nervous system activity, thereby reducing heart rate and myocardial oxygen demand. The third class consists of mineralocorticoid receptor antagonists (MRA), which inhibit the effects of aldosterone and reduce sodium and water retention [1,2]. Collectively, this conventional triple therapy mitigates cardiac remodeling. Landmark clinical trials have demonstrated that RASi significantly reduce the incidence of cardiovascular (CV) death and heart failure hospitalization (HFH) [3,4,5,6,7]. In parallel, BB and MRA have been shown to reduce all-cause mortality and HFH [8,9,10,11,12]. The fourth class is the sodium–glucose cotransporter 2 inhibitors (SGLT2i), a recent addition to heart failure treatment guidelines. Originally developed as antihyperglycemic agents, this class exerts its effects by inhibiting glucose reabsorption in the proximal tubule through selective blockade of sodium–glucose co-transporter 2. Subsequent landmark trials have provided evidence supporting the use of SGLT2i in patients with HFrEF, regardless of the presence or absence of concomitant diabetes mellitus. The DAPA-HF and EMPEROR-Reduced studies demonstrated that dapagliflozin and empagliflozin significantly reduced the composite endpoint of CV death and HFH. Notably, dapagliflozin was also shown to reduce the risk of death from cardiovascular causes. These findings have led to the incorporation of dapagliflozin and empagliflozin into current clinical guidelines for the treatment of HFrEF, regardless of diabetes status [13,14].

Despite strong evidence supporting the clinical benefits of GDMT in chronic HFrEF, including significant reductions in mortality and hospitalizations, previous studies have shown that the effectiveness of conventional triple therapy (RASi, BB and MRA) is markedly enhanced when each drug is administered at target doses (TD) (≥50% TD) [15]. Prior investigations in the United States, Europe, and Asia have demonstrated that only approximately one-fourth of HFrEF patients receive conventional triple therapy at TD. Moreover, more than half of patients are prescribed RASi and BB at low-dose, defined as <50% TD [15,16,17]. Data from the ASIAN-HF registry, which encompasses multiple countries across Asia, revealed that only 32% of patients received RASi and 26.8% received BB at ≥50% TD, whereas 46.5% received RASi and 48.9% received BB at only 0–24% TD [17]. Consistent with these findings, data from Thailand similarly indicate that only 16% of patients received BB at ≥50% TD, and 52.8% were treated at 0–24% TD [18]. Taken together, international and Thai data underscore that the majority of HFrEF patients are treated with inadequate dose of RASi and BB, thereby limiting the full therapeutic potential of these agents.

For patients with chronic HFrEF, treatment with BB and RASi is typically initiated at a low-dose and subsequently titrated upward in small increments until the target dose is reached. This stepwise titration process is one of the key factors contributing to the frequent use of low-dose in clinical practice. In contrast, the use of SGLT2i in patients with chronic HFrEF generally circumvents this issue, as these agents are prescribed at a single standardized dose in which the initial and target dose are identical. Consequently, dose titration is unnecessary, helping to ensure that patients consistently receive the target dose. Post hoc analysis suggests that HFrEF patients receiving RASi and/or BB at low-dose in combination with SGLT2i, may achieve clinical outcomes, defined as the composite of CV death and heart failure hospitalization, comparable to those observed in patients receiving high-dose RASi and BB without SGLT2i therapy [19,20]. However, direct comparative data between these two groups remains lacking. A previous study conducted in an intensive heart failure clinic at one of our current study sites (Chiangrai Prachanukroh Hospital) reported that the cardiovascular mortality rates at 12 months were 16.42%, while the rehospitalization from heart failure was 24–27%, despite more than 50% TD of GDMT being achieved in those patients (88% of patients received RASi at ≥50% TD, 98% of patients received BB at ≥50% TD and 88% of patients received MRA at ≥50% TD) and only 10.45% receiving SGLT2i [21]. Heart failure management in Thailand faces ongoing challenges, particularly in the titration of RASi and BB to achieve ≥50% TD. In addition, access to SGLT2i remains limited for a subset of heart failure patients. Based on the aforementioned data, a substantial proportion of patients with HFrEF are unable to access SGLT2i. In this group, physicians attempt to titrate RASi and BB to achieve ≥50% of TD. In contrast, some patients cannot tolerate RASi or BB at doses ≥50% of TD, yet remain eligible for treatment with SGLT2i.

This highlights the critical need for evidence-based strategies to ensure appropriate pharmacologic therapy tailored to individual patient profiles, which is essential for optimizing outcomes and maximizing the therapeutic benefits of chronic HFrEF management. This study aimed to compare clinical cardiovascular outcomes, particularly CV death and HFH, in routine clinical practice among patients with HFrEF treated with low-dose RASi and/or BB plus MRA in combination with SGLT2i, versus those receiving high-dose RASi and beta-blockers plus MRA without SGLT2i.

## 2. Materials and Methods

### 2.1. Study Design and Setting

This retrospective observational study used electronic medical records of patients with chronic heart failure who attended outpatient clinics at Chiangrai Prachanukroh Hospital (Chiang Rai Province) and Naresuan University Hospital (Phitsanulok Province), Thailand. Ethical approval was obtained from the Human Research Ethics Committee of Chiangrai Prachanukroh Hospital (CRH), protocol number EC CRH 082/66 In, approved on 11 September 2023. This approval also granted permission to access and collect data from Naresuan University Hospital (NUH).

### 2.2. Participants

#### 2.2.1. Sample Size

Sample size was calculated using the [artbin] command in Stata version 14.0 based on a non-inferiority design [22,23]. Expected event rates of 17% and 18% for the exposed cohort and reference cohort respectively were derived from post hoc analysis of the DAPA-HF trial [19]. A non-inferiority margin of 13% was adopted from the VALIANT trial [24]. Assuming a one-sided alpha of 0.025, 80% power, and a 2:1 allocation ratio, the required total sample size was 321 participants (214 in the reference cohort and 107 in the exposed cohort). The calculation was based on asymptotic normal approximations, which are suitable for planning trials with binary endpoints [22,23].

#### 2.2.2. Inclusion and Exclusion Criteria

This study included all patients aged ≥18 years with a history of chronic heart failure who attended outpatient clinics between 31 March 2018 and 31 March 2024. Eligibility was defined using the International Classification of Diseases, Tenth Revision (ICD-10 codes), including I50 (heart failure), I25.0 (atherosclerotic cardiovascular disease), I25.1 (atherosclerotic heart disease), I25.2 (old myocardial infarction), I25.5 (ischemic cardiomyopathy), I42.0 (dilated cardiomyopathy), I42.6 (alcoholic cardiomyopathy), and I42.9 (unspecified cardiomyopathy), and a documented LVEF <40%. HFrEF Patients who subsequently developed a diagnosis of Heart Failure with improved Ejection Fraction (HFimpEF) were also eligible. All patients were required to have received guideline-recommended conventional triple therapy at stable doses for at least 3 months [25].

**Figure 1:**
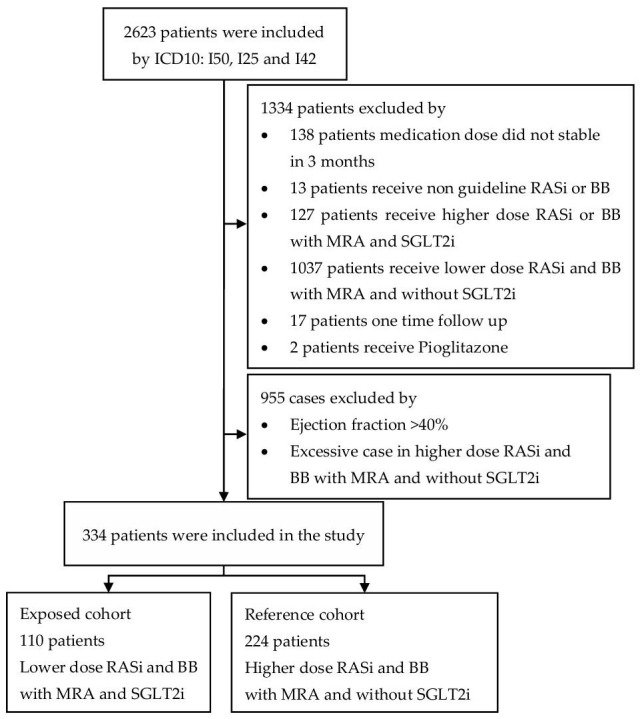
Flow chart of participants included in the study.

Patients were excluded if they had only a single outpatient visit or received RASi or BB not recommended by heart failure guidelines (e.g., metoprolol tartrate, atenolol, and propranolol). Specifically, patients receiving ≥50% TD of RASi and BB combined with SGLT2i were excluded, as were those receiving spironolactone at a dose lower than 25 mg. Additionally, patients receiving <50% of the recommended dose without SGLT2i were also excluded. Furthermore, exclusion criteria included SGLT2i treatment for less than one month, or the concomitant use of pioglitazone or non-dihydropyridine calcium channel blockers. In total, 334 patients were included in the study (Figure 1).

### 2.3. Definition of Terms

The conventional triple therapy doses used in the study were based on heart failure clinical practice guideline recommendations. Participants were stratified by dose intensity into two categories: high-dose (≥50% of the guideline target dose) and low-dose (<50% of the guideline target dose) [15,16,17,18,19,20].

The exposed cohort included patients who received SGLT2i with at least one of RASi or BB prescribed at a low-dose (<50% of the target dose) plus MRA. This treatment pattern is referred to as SGLT2i with low-dose conventional triple therapy. The reference cohort consisted of patients receiving high-dose conventional triple therapy defined as high-dose (≥50% of the target dose) of both RASi and BB plus MRA but without SGLT2i.

Comorbidities were defined as non-heart failure conditions identified by ICD-10 codes, including hypertension (I10–I15), ischemic heart disease (I20–I25), atrial fibrillation (I48), diabetes mellitus (E10–E14), chronic obstructive pulmonary disease (J44), asthma (J45), and chronic kidney disease (N18).

Concomitant medications were defined as non-GDMT cardiovascular medication prescribed for patients. These included digoxin, hydralazine, isosorbide dinitrate, isosorbide mononitrate, ivabradine, trimetazidine, furosemide, hydrochlorothiazide, dihydropyridine and non-dihydropyridine calcium channel blockers (CCB), pioglitazone, and non-steroidal anti-inflammatory drugs (NSAID).

Hospitalization was defined as a physician-directed inpatient admission for heart failure, requiring treatment in a hospital.

CV death was defined based on a physician-diagnosed primary cause of death as documented in the hospital’s medical records. Cases lost to follow-up were censored at the date of the last contact

### 2.4. Data Collection

Patient data were collected from electronic medical records between 31 March 2018, and 31 March 2024. The outpatient dataset included the following patient characteristics: age, sex, date of outpatient visit, New York Heart Association (NYHA) functional class, LVEF, vital signs (blood pressure, heart rate), body weight, height, comorbidities classified by ICD-10 codes, prescribed cardiovascular medications, and laboratory results (serum potassium and estimated glomerular filtration rate [eGFR]). Inpatient data included date of hospital admission, principal diagnosis, and cardiovascular medications prescribed at discharge.

### 2.5. Study Objectives and Outcomes

This study aimed to compare clinical outcomes between the exposed cohort (SGLT2i with low-dose conventional triple therapy) and the reference cohort (high-dose conventional triple therapy). The primary outcome of this study was a composite endpoint consisting of cardiovascular death and heart failure hospitalization. Secondary outcomes included each individual component of the primary outcome, specifically cardiovascular death and heart failure hospitalization.

### 2.6. Statistical Analysis

Data was pre-processed using Microsoft Excel (Microsoft 365, private license) and analyzed with STATA software (version 14.0; Stata Corp, College Station, TX, USA; licensed to Faculty of Pharmacy, Chiang Mai University).

Baseline characteristics were summarized as mean and standard deviation (SD) or median with interquartile range (IQR) for continuous variables, and as counts with percentages for categorical variables. The Shapiro–Wilk test was used to assess the normality of continuous variables. Between-group differences were examined using the independent *t*-test or Wilcoxon rank-sum test for continuous data, and Fisher’s exact test for categorical data. The study was designed as a non-inferiority framework. The non-inferiority margin was prespecified at a hazard ratio (HR) of 1.13, based on prior evidence [24]. Non-inferiority was established if the upper limit of the one-sided 97.5 percent confidence interval for the comparison between the exposed cohort and the reference cohort was below 1.13.

To minimize confounding prior to outcome analysis, inverse probability of treatment weighting (IPTW) based on propensity scores was applied to construct a weighted cohort with improved covariate balance. Covariate balance was assessed using absolute standardized differences (ASD), and covariates with ASD > 10% between groups were considered imbalanced. These imbalanced covariates, together with variables showing *p* < 0.20 in univariable Cox analyses of the weighted cohort, were subsequently included in the multivariable Cox proportional hazards model. This approach represents a covariate-adjusted IPTW analysis, conceptually related to double robust methods. Kaplan–Meier curves were generated from the crude data to illustrate the cumulative incidence of outcomes, and between-group differences were assessed using the log-rank test. Cox proportional hazards regression was applied to estimate hazard ratios (HRs) with 95% confidence intervals (CIs).

Final results were reported as HR with corresponding 95% CIs. Non-inferiority was assessed by comparing the upper bound of the 95% CI for HR against the prespecified non-inferiority margin. The primary analysis in the present study was conducted using a Cox proportional hazards model and expressed as a hazard ratio (HR), which is the standard approach for time-to-event outcomes. The proportional hazards assumption was rigorously verified using Schoenfeld residuals (*p* = 0.611), confirming the appropriateness of the cox model for this analysis.

## 3. Results

### 3.1. Patient Baseline Characteristics and Medication Dosing

A total of 2623 outpatient records were collected from the database between 31 March 2018, and 31 March 2024. After applying the study’s inclusion and exclusion criteria, 334 patients were ultimately recruited into the final cohort. We enrolled 263 patients from Chiangrai Prachanukroh Hospital and 71 patients from Naresuan University Hospital; 298 patients (89.2%) were classified as having NYHA functional class I or II. The most prevalent comorbidities included hypertension, ischemic heart disease (IHD), atrial fibrillation (AF), diabetes mellitus (DM), and chronic kidney disease (CKD). The patients were divided into two comparative groups, with 110 patients in the exposed cohort, and 224 patients in the reference cohort. Baseline characteristics are presented in Table 1. Baseline characteristics were significantly different between the two groups for various variables before adjustment. Compared with the control group, patients in the exposed cohort had a higher prevalence of comorbid conditions, including hypertension, ischemic heart disease, atrial fibrillation, and diabetes mellitus. They also exhibited lower average blood pressure and eGFR. Additionally, a greater proportion of patients in this group received ASA, furosemide, ivabradine and trimetazidine. Patients in the exposed cohort had a mean age of 63.5 years. The majority (66 (60.0%) patients) were covered under the civil servant medical benefit scheme (CSMBS). BB was prescribed in 96 patients (87.3%), with a median dose of 25.00% TD. RASi was used in 102 patients (92.7%), with a median dose of 33.33% TD. Among SGLT2i, dapagliflozin was prescribed in 88 patients (80.0%) and empagliflozin in 22 patients (20.0%). The reference cohort had a mean age of 61.0 years. The majority were covered under the universal coverage scheme (UCS), accounting for 193 (86.2%) patients. The median dose of BB was 100% TD and that of RASi was 75% TD. The median MRA (spironolactone) dose was 25 mg in both groups (73.6% of patients in the exposed cohort and 69.6% in the reference cohort), with no significant difference between the two cohorts (*p* = 0.666).

After balancing patient characteristics using Inverse Probability of Treatment Weighting (IPTW), variables with an absolute standardized difference (ASD) exceeding 10% included NYHA functional class, systolic blood pressure, LVEF, case type, atrial fibrillation, estimated glomerular filtration rate (eGFR), and the administration of aspirin (ASA), NSAIDs, furosemide, hydralazine, isosorbide mononitrate (ISMN), ivabradine, and trimetazidine in Table 2.

Consequently, these imbalanced covariates, along with other potential predictors identified in the weighted univariable Cox proportional hazards models with a *p*-value < 0.20, were considered for further analysis. These included age, body mass index (BMI), study site, systolic blood pressure, diastolic blood pressure, ejection fraction, eGFR, use of furosemide, hydralazine, and a history of hypertension, ischemic heart disease, and atrial fibrillation, shown in Table 3. Ultimately, these selected variables were included in the final multivariable weighted Cox proportional hazards analysis.

### 3.2. Association Between Treatment Strategy and Cardiovascular Death or Heart Failure Hospitalization

**Figure 2:**
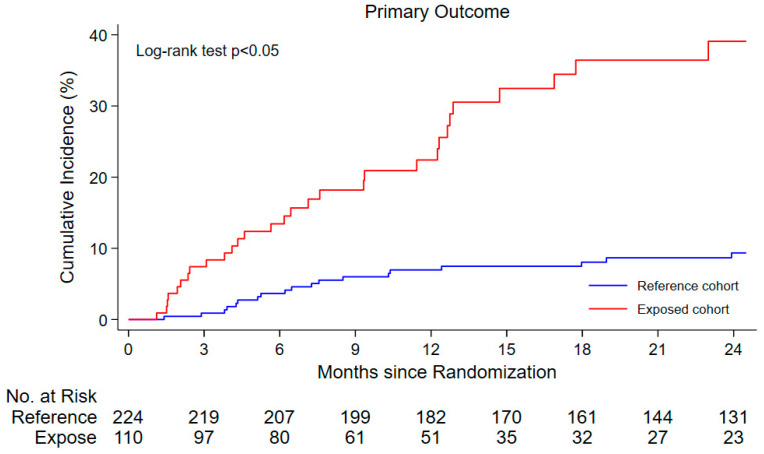
The Kaplan–Meier curve for survival composite outcome.

The mean follow-up duration was 1.2 years in the exposed cohort and 2.5 years in the reference cohort. A total of 60 patients experienced the composite outcome of CV death and HFH. The composite outcome occurred more frequently in the exposed cohort (32 patients, crude event rate 24.38 per 100 patient-years) compared to the reference cohort (28 patients, 4.99 per 100 patient-years). Similarly, crude event rates were higher in the exposed group for CV death (6.09 vs. 0.89 per 100 patient-years) and heart failure hospitalization (19.5 vs. 5.17 per 100 patient-years), respectively. Figure 2 shows a higher incidence of the composite outcome in the exposed cohort.

After applying IPTW and conducting multivariable Cox proportional hazards analysis, patients in the exposed cohort showed a significantly higher risk of experiencing the composite outcome (adjusted HR 4.10, 95% CI 2.07–8.13, *p* < 0.001). Non-inferiority could not be established. Based on the secondary outcome analysis using a multivariable cox proportional hazards model with IPTW, the two treatment cohorts showed no significant difference in CV death (adjusted HR 6.40, 95% CI 0.89–46.19, *p* = 0.065). However, the exposure cohort had a higher rate of HFH (adjusted HR 4.47, 95% CI 2.20–9.06, *p* < 0.001) (Table 4).

## 4. Discussion

This study was based on retrospective data collection beginning in 2018, during which SGLT2i had not yet been approved in Thailand for the treatment of chronic heart failure. The primary objective was to evaluate whether the exposed cohort could achieve comparable clinical effectiveness to the reference cohort. Within this comparative context, we employed a non-inferiority framework. However, non-inferiority was not demonstrated, as the exposed cohort exhibited a higher incidence of the composite outcome compared to the reference cohort. These findings differ from post hoc analyses of the DAPA-HF and EMPEROR-Reduced trials, which reported comparable incidences of composite outcomes between patients receiving low-dose of conventional triple therapy combined with a SGLT2i and those receiving high-dose conventional triple therapy [19,20]. Differences in patient characteristics and treatment accessibility may partly explain the divergence from DAPA-HF and EMPEROR-Reduced. Although propensity scores with IPTW were applied in our study to adjust baseline clinical characteristics at study entry in order to enhance comparability between the two groups, and a multivariable Cox proportional hazards model was used, patients in the exposed cohort still exhibited a higher incidence of the composite outcome compared with those in the reference cohort. Several systematic reviews and network meta-analyses suggest that combination therapy with multiple guideline-recommended medications, especially ACEI + BB + MRA + SGLT2i or ARNI + BB + MRA, reduces the incidence of the primary composite outcome more effectively than partial regimens. Patients receiving ACEI + BB + MRA + SGLT2i showed a 10–25% reduction in composite outcomes and an 8–25% reduction in heart failure hospitalization compared to those not receiving SGLT2i. However, these findings are based on indirect comparisons and do not account for actual medication doses, limiting conclusions about dose–response relationships [26,27,28].

Although the primary publications of the DAPA-HF and EMPEROR-Reduced trials did not include detailed neurohormonal blockade (NHB) doses, post hoc analyses from both studies did provide some data. In the DAPA-HF trial, 49.5% of patients received BB at more than 50% TD, while 35.8% received RASi at over 50% TD. In the EMPEROR-Reduced trial, the corresponding figures were 51.7% and 46.4%, respectively. Based on data from both trials, the average dosing of BB and RASi was likely close to 50% TD [19,20]. Importantly, while these trials demonstrated benefit from SGLT2i regardless of background dose, a notable finding from the EMPEROR-Reduced post hoc analysis revealed that patients receiving RASi at less than 50% TD appeared less likely to derive the full benefit from empagliflozin in reducing hospitalization risk [20]. The NHB is an important component of heart failure management, particularly the use of NHB at target dose. High-dose NHB appears to confer greater clinical benefit than low-dose [15,16,17]. In contrast, patients in our study who received SGLT2i had substantially lower median dose, i.e., 25.00% TD for BB and 33.33% TD for RASi, representing a very low-dose of conventional triple therapy. This may have attenuated the clinical benefit of SGLT2i in reducing the composite outcome. Conversely, patients in the reference cohort who received high-dose BB and RASi (median 100% TD and 75% TD, respectively) with target dose of MRA but without SGLT2i, were treated at very high-dose. This may partly explain the superior composite outcome profile in the reference cohort and the divergence from findings in the landmark trials. A sensitivity analysis was conducted by excluding patients who received very low doses (less than 25% of the target dose) of any conventional triple therapy. This refined the cohort to 300 cases. Interestingly, after this exclusion, the Hazard Ratio (HR) for the primary outcome remained comparable to the original estimate and increased slightly to 4.89 (95% CI: 2.07–11.56, *p* < 0.001). These findings reinforce the robustness of our primary analysis and clearly demonstrate that the high-risk profile observed in the exposed cohort remains consistent, even when foundational heart failure treatments are titrated to more clinically significant levels. Our findings should be interpreted within the context of real-world clinical complexity. Recent evidence in HFrEF populations suggests that the clinical benefits of contemporary therapies are closely linked to dose–response patterns and specific patient phenotypes [29,30].

Data from prior studies with patient characteristics similar to our current study reported fewer cardiovascular deaths or heart failure admissions in HFrEF patients receiving conventional GDMT. Specifically, the PARADIGM-HF trial reported an event rate of 26.5 in the group receiving enalapril at 20 mg/day over a median follow-up of 27 months, which means an event rate of 11.8 per 100 patient-years. Furthermore, the HEAAL study reported an event rate of 9.3 per 100 patient-years in the group receiving losartan 150 mg and 10.7 per 100 patient-years in the group receiving losartan 50 mg. In contrast, studies within Thailand have previously reported a higher burden of events. The ASIAN-HF registry encompasses both heart failure with reduced ejection fraction (HFrEF) and heart failure with preserved ejection fraction. The cardiovascular (CV) death rate among HFrEF patients was approximately 8.0 per 100 patient-years [31], whereas in our exposed cohort, the IPTW-adjusted CV death rate was 6.4 per 100 patient-years (8.03 after IPTW). Specifically, a study on BB use in HFrEF patients in Thailand reported a composite outcome of all-cause mortality and hospitalization at a rate of 49.5 per 100 patient-years. Notably, that study did not report the analysis of beta-blocker and RASi dosages as a percentage of their respective target doses. A previous study conducted at Chiangrai Prachanukroh Hospital, involving HFrEF patients receiving intensive treatment and followed for one year, found CV death rates of 16.42 per 100 patient-years and rehospitalization from heart failure at 24.24 per 100 patient-years, despite more than 50% TD of GDMT with 10% received SGLT2i [21]. Although the exposed cohort in our study exhibited a higher incidence of the composite outcome compared with the reference cohort, the event rates for the composite endpoint, CV death, and heart failure hospitalization were lower than those reported in previous Thai studies.

During the initial data collection period of this study, SGLT2i use in Thailand was primarily restricted to diabetes mellitus indication and had not yet been officially approved for HFrEF, even following the publication of the DAPA-HF and EMPEROR-Reduced trial results. Consequently, patients in the cohort receiving SGLT2i with low-dose NHB were predominantly those diagnosed with HFrEF and concomitant diabetes mellitus. This resulted in a significantly higher prevalence of diabetes mellitus as a comorbidity in the exposed group. Furthermore, this cohort also presented with a higher burden of other high-risk conditions, including hypertension, atrial fibrillation, and ischemic heart disease. The higher prevalence of these comorbidities is a critical factor, as managing these conditions is deemed essential for reducing morbidity and mortality in heart failure patients. These conditions are specifically addressed in contemporary heart failure guidelines, underscoring their substantial prognostic impact. This baseline burden of comorbidities likely contributed to the poorer clinical outcomes observed in this patient group [1,2]. Furthermore, differences in disease pathophysiology and severity persisted between the cohorts, as suggested by baseline data on NYHA functional class, principal cause of heart failure, and blood pressure. Even after adjustment using IPTW and the multivariable Cox proportional hazards model, these remaining differences likely represent residual confounding that influenced treatment response and subsequent clinical outcomes. Evidence consistently indicates that heart failure patients with non-ischemic etiology respond better to medical therapy, present with fewer comorbidities, and experience lower rates of cardiovascular death CV death and HFH compared to those with ischemic etiology [32]. Even after adjusting baseline characteristics, more patients in the exposed cohort received furosemide in addition to GDMT, which aligns with HF guidelines recommending diuretics in patients who have fluid retention to relieve congestion, improve symptoms, and prevent worsening HF. Consequently, this data suggests that patients in the exposed cohort may have more unstable disease status and a higher likelihood of HF worsening compared to the reference cohort, who received less Furosemide, reflecting a lower risk of HF worsening in that group [1,2].

Additionally, the median BMI of patients in both groups was approximately 23.5 kg/m^2^. Previous research examining the relationship between BMI and all-cause mortality in chronic heart failure has suggested that the lowest risk is observed among patients with a BMI between 25 and 40 kg/m^2^ [33]. Therefore, patients in this study may have had a higher baseline risk compared to those in the DAPA-HF and EMPEROR-Reduced trials, where the average BMI was reported at 28 kg/m^2^. However, this does not suggest that patients should gain weight, as the observed association may be confounded by other factors. Clinical care should remain individualized and guided by evidence-based risk modification.

Recent evidence highlights the extensive pleiotropic effects of SGLT2 inhibitors beyond diuresis and glucose lowering. Notably, these agents exert significant anti-arrhythmic benefits, which are vital in reducing the burden of atrial and ventricular arrhythmias in heart failure patients [34]. In our study, despite these well-documented benefits, the exposed cohort which featured a markedly higher baseline prevalence of atrial fibrillation (ASD 0.282) still exhibited a significantly higher event rate (HR 4.10). This divergence suggests that in real-world clinical practice, the profound baseline disease severity and high-risk profile may temporarily outweigh the pleiotropic benefits of SGLT2 inhibitors, particularly when background conventional triple therapy is maintained at very low dose (nearly 25% of target).

Furthermore, the observed outcomes may be attributed to a cluster of residual confounding that persists despite a rigorous doubly robust approach using IPTW and Cox proportional hazards models. This confounding cluster encompasses baseline disease severity and a high-risk clinical profile, including frailty, prolonged disease duration, and a potentially intolerant phenotype toward the up-titration of foundational therapies. When these factors aggregate, they create a complex confounding environment that may temporarily obscure the therapeutic benefits of SGLT2 inhibitors, particularly in a real-world setting where background conventional triple therapy remains at a very low dose.

Therefore, patients with heart failure in Thailand should receive conventional GDMT at target dose whenever feasible, with the addition of SGLT2i as appropriate. Several studies, including clinical trials and meta-analyses, have supported the use of all four guideline-recommended drug classes for chronic heart failure. However, no prior reports have specifically evaluated outcomes based on adherence to target dose, particularly regarding individual drug titration. This study is the first to assess the impact of heart failure pharmacotherapy in a real-world Thai population with varying limitations in drug access, dose, and regimen composition. The findings may help inform treatment planning for patients with restricted access to certain medications.

While the benefits of SGLT2i are robust, very low dosing of conventional triple therapy may attenuate overall clinical optimization. This underscores that SGLT2i should be used in conjunction with efforts to up-titrate conventional triple therapy whenever feasible, rather than prioritizing dose escalation of conventional therapy alone without the addition of SGLT2i in patients who are eligible to receive it. However, in patients who are unable to receive SGLT2i, efforts to titrate conventional triple therapy to the maximally tolerated target dose remain essential, as this approach has been clearly demonstrated to provide significant clinical benefit.

This study aligns with the United Nations Sustainable Development Goal (SDG) 3, particularly Targets 3.4 and 3.8, and contributes to SDG 10 on Reduced Inequalities. It provides evidence to inform treatment strategies aimed at reducing cardiovascular death and heart failure hospitalizations, thereby supporting SDG Target 3.4. The findings underscore the importance of optimizing guideline-directed medical therapy, which is central to achieving universal health coverage and ensuring access to safe, effective, and essential medicines in line with SDG Target 3.8. Conducted in Thailand, this study offers context-specific evidence for resource-limited and middle-income settings where SGLT2 inhibitors remain costly, supporting equitable, evidence-based care and helping to reduce disparities related to financial and resource constraints in accordance with SDG 10.

This study has several limitations. First, there is a potential for selection bias, as the exposed cohort likely represents a higher-risk or intolerant phenotype of patients who could not undergo further up-titration of foundational therapies. Second, as a retrospective analysis, some medical records were incomplete, and certain data such as patient or caregiver adherence, reasons for dose adjustments (for hemodynamic instability, worsening renal function, or other side effects), reasons for treatment discontinuation, period for up-titration, documentation of furosemide use under flexible diuretic guidelines, and HF status (severity and HF progress, repeat EF measurement, laboratory parameters in HF patients) were unavailable. Perhaps this missing data may have influenced the interpretation of outcomes. Future studies should adopt a prospective design to capture additional clinical details, particularly the rationale for medication discontinuation and barriers to dose up-titration. Moreover, limited access to SGLT2i remains a challenge in Thailand. As access improves, further analyses in larger populations will be valuable to better define the optimal sequencing of GDMT when titration to target doses faces real-world constraints, ultimately guiding more effective treatment strategies.

Our study underscores the importance of titrating GDMT to the highest tolerated target dose, highlighting that effective heart failure management requires not only the use of all four GDMT classes but also optimization of each agent whenever feasible.

## 5. Conclusions

In this study of patients with HFrEF, SGLT2 inhibitor combined with low-dose conventional triple therapy did not demonstrate comparable clinical outcomes to high-dose conventional triple therapy with respect to cardiovascular outcomes, defined as the composite of cardiovascular death and heart failure hospitalization. This finding appears particularly pronounced in this cohort, which presented with a notably higher baseline burden of disease severity. Further investigation is warranted to clarify these findings and their implications for the management of HFrEF.

## Figures and Tables

**Table 1:** Patient characteristics at baseline.

Characteristic	Exposed Cohort (*n* = 110)	Reference Cohort (*n* = 224)	*p*-Value
Sex			
Male—no. (%)	73 (66.36)	137 (61.16)	0.400
Female—no. (%)	37 (33.64)	87 (38.84)	
Age—yr, median (IQR)	63.5 (72–57)	61 (49–67)	<0.001
Body mass index, median (IQR)	23.54 (21.05–27.77)	23.52 (21.19–26.56)	0.971
Health insurance—no. (%)			
UCS	44 (40)	193 (86.16)	<0.001
CSMBS	66 (60)	31 (13.84)	
Study site—no. (%)			
CRH	67 (60.91)	196 (87.50)	<0.001
NUH	43 (39.09)	28 (12.50)	
NYHA functional class—no. (%)			
I–II	93 (84.55)	205 (91.52)	0.061
III	17 (15.45)	19 (8.48)	
Case type			
Old (more than 6 months)	27 (24.55)	137 (61.16)	<0.001
New	83 (75.45)	87 (38.84)	
Pulse rate—beats/min, mean ± SD	79.75 ± 15.67	77.05 ± 12.97	0.121
Systolic blood pressure—mm Hg, mean ± SD	118.59 ± 20.50	126.13 ± 22.44	0.003
Diastolic blood pressure—mm Hg, mean ± SD	69.90 ± 14.06	73.25 ± 14.73	0.045
Left ventricular ejection fraction—%	25.99 ± 7.41	25.67 ± 7.11	0.706
Principal cause of heart failure—no. (%)			
Ischemic (I25)	19 (17.27)	21 (9.38)	0.048
Nonischemic (I42, I50)	91 (82.73)	203 (90.62)	
Medical history—no. (%)			
Hypertension	40 (36.36)	37 (16.52)	<0.001
Ischemic heart disease	18 (16.36)	10 (4.46)	0.001
Atrial fibrillation	15 (13.64)	12 (5.36)	0.017
Diabetes mellitus	36 (32.73)	11 (4.91)	<0.001
Chronic kidney disease	9 (8.18)	7 (3.13)	0.056
Serum Potassium	4.12 ± 0.54	4.20 ± 0.57	0.258
Estimated glomerular filtration rate			
Median—mL/min/1.73 m^2^ (IQR)	62.95 (46–84.11)	70.5 (54.00–92.5)	0.018
Rate of eGFR < 60 mL/min/1.73 m^2^—no. (%)	46 (41.82)	77 (34.38)	0.187
Conventional triple therapy dose			
Beta-blockers—%TD, median (IQR)	25 (25–50)	100 (100–100)	<0.001
RAS inhibitors—%TD, median (IQR)	33.33 (25–50)	75 (66.67–100)	<0.001
MRA (spironolactone)—mg, median (IQR)	25 (25–25)	25 (25–25)	0.666
Medication—no. (%)			
Beta-blockers			<0.001
No	14 (12.73)	0 (0.00)	
Bisoprolol	39 (35.45)	33 (14.73)	
Carvedilol	57 (51.82)	189 (84.38)	
Nebivolol	0 (0.00)	2 (0.89)	
RAS inhibitors			<0.001
No	8 (7.27)	0 (0.00)	
ACEI	43 (39.09)	101 (45.09)	
ARB	30 (27.27)	113 (50.45)	
ARNi	29 (26.36)	10 (4.46)	
MRA (Spironolactone)	110 (100.00)	224 (100.00)	
SGLT2 inhibitors			
No	0 (0.00)	224 (100.00)	
Dapagliflozin	88 (80.00)	0 (0.00)	
Empagliflozin	22 (20.00)	0 (0.00)	
ASA	60 (54.55)	95 (42.41)	0.047
Digoxin	11 (10.00)	36 (16.07)	0.180
Furosemide	102 (92.73)	177 (79.02)	0.001
Hydralazine	8 (7.27)	15 (6.70)	0.822
ISMN	18 (16.36)	21 (9.38)	0.071
Ivabradine	17 (15.45)	8 (3.57)	<0.001
Trimetazidine	10 (9.09)	2 (0.89)	<0.001
NSAIDs	14 (12.73)	8 (3.57)	0.004

Exposed cohort: SGLT2i with low-dose conventional triple therapy group, reference cohort: high-dose conventional triple therapy. Abbreviations: UCS, universal coverage scheme; CSMBS, civil servant medical benefit scheme; CRH, Chiangrai Prachanukroh Hospital; NUH, Naresuan University Hospital; eGFR, estimated glomerular filtration rate; TD, target dose; RAS inhibitors, Renin-Angiotensin System inhibitors; ACEI, Angiotensin-Converting Enzyme Inhibitors; ARB, Angiotensin Receptor Blockers; ARNi, Angiotensin Receptor-Neprilysin Inhibitor; MRA, Mineralocorticoid Receptor Antagonists; SGLT2 inhibitors, Sodium-Glucose Cotransporter 2 inhibitors; ASA, Aspirin at any dose; ISMN, isosorbide mononitrate; NSAIDs, Non-Steroidal Anti-Inflammatory Drugs.

**Table 2:** Balance of patient characteristics after IPTW.

Characteristic	Exposed Cohort (*n* = 110)	Reference Cohort (*n* = 224)	Standardized Difference
Sex			
Male	68.4%	64.6%	0.080
Female	31.6%	35.4%	−0.080
Age, mean	58.87	58.95	−0.006
Body mass index, mean	24.44	24.38	0.070
Health insurance			
UCS	62.6%	66.5%	−0.083
CSMBS	37.4%	33.5%	0.083
Study site			
CRH	76.1%	75.5%	0.013
NUH	23.9%	24.5%	−0.013
NYHA functional class			
I–II	86.6%	90.4%	−0.117
III	13.4%	9.6%	0.117
Pulse rate, mean	80.07	79.31	0.053
Systolic blood pressure, mean	116.25	126.20	−0.463
Diastolic blood pressure, mean	72.41	70.97	0.100
Left ventricular ejection fraction, mean	22.79	24.48	−0.232
Case type			
Old case (more than 6 months)	39.2%	46.9%	−0.156
New case	60.8%	53.1%	0.156
Principal cause of heart failure			
Ischemic	9.3%	7.8%	0.056
Nonischemic	90.7%	92.2%	−0.056
Medical history			
Hypertension	34.1%	30.6%	0.075
Ischemic heart disease	8.2%	8.7%	−0.016
Atrial fibrillation	21.8%	11.4%	0.282
Diabetes mellitus	17.6%	17.3%	0.009
Chronic kidney disease	5.3%	4.7%	0.027
Estimated glomerular filtration rate, mean	66.58	70.57	−0.161
Serum Potassium, mean	4.17	4.20	−0.055
Concomitant medication			
ASA	44.5%	50.4%	−0.118
NSAIDs	9.1%	6.3%	0.106
Digoxin	14.3%	12.6%	0.051
Furosemide	94.0%	82.8%	0.355
Hydralazine	14.3%	7.2%	0.232
ISMN	20.2%	9.6%	0.301
Ivabradine	13.7%	3.2%	0.384
Trimetazidine	6.2%	0.7%	0.305

Exposed cohort: SGLT2i with low-dose conventional triple therapy group, reference cohort: high-dose conventional triple therapy. Abbreviations: UCS, universal coverage scheme; CSMBS, civil servant medical benefit scheme; CRH, Chiangrai Prachanukroh Hospital; NUH, Naresuan University Hospital; ASA, Aspirin at any dose; NSAIDs, Non-Steroidal Anti-Inflammatory Drugs; ISMN, isosorbide mononitrate.

**Table 3:** Univariable Cox proportional hazards analysis with IPTW for patient characteristics.

Characteristics	HR for Composite Outcome (95% CI)	*p*-Value	HR for Composite Outcome After IPTW (95% CI)	*p*-Value	HR for Dead After IPTW (95% CI)	HR for Hospitalization After IPTW (95% CI)
SGLT2i	4.31 (2.53–7.33)	<0.001	4.77 (2.14–10.61)	<0.001	2.22 (0.41–2.07)	6.79 (3.40–13.57)
Sex—Female	1.10 (0.65–1.84)	0.723	0.68 (0.32–1.42)	0.302	0.29 (0.07–1.23)	0.96 (0.41–2.27)
Age	1.04 (1.02–1.06)	0.001	1.04 (1.05–1.07)	0.023	1.09 (1.04–1.15)	1.02 (0.99–1.05)
Body mass index	0.95 (0.89–1.01)	0.089	0.93 (0.87–0.98)	0.012	0.92 (0.81–1.05)	0.92 (0.86–0.99)
Health insurance—CSMBS	1.35 (0.79–2.31)	0.270	0.77 (0.35–1.71)	0.525	1.42 (0.28–7.31)	0.64 (0.30–1.37)
Study site–NUH	1.32 (0.71–2.45)	0.380	0.60 (0.28–1.31)	0.199	0.28 (0.05–1.68)	0.78 (0.34–1.79)
New case in 6 months	1.91 (1.14–3.21)	0.015	0.74 (0.36–1.54)	0.426	1.64 (0.26–10.44)	0.53 (0.26–1.06)
Principal cause of HF – Nonischemic	1.03 (0.44–2.40)	0.946	1.25 (0.47–3.28)	0.654	0.93 (0.16–5.30)	1.51 (0.47–4.92)
NYHA functional class	1.13 (0.49–2.64)	0.772	0.93 (0.35–2.48)	0.885	0.21 (0.02–1.81)	1.22 (0.43–3.49)
Pulse rate	1.02 (1.00–1.03)	0.099	1.00 (0.97–1.03)	0.845	1.01 (0.93–1.08)	1.00 (0.97–1.02)
Systolic blood pressure	0.98 (0.98–1.00)	0.045	0.98 (0.97–1.00)	0.022	0.99 (0.96–1.03)	0.98 (0.97–0.99)
Diastolic blood pressure	0.98 (0.96–1.00)	0.065	0.98 (0.95–1.01)	0.130	0.92 (0.88–0.96)	1.00 (0.98–1.02)
LVEF	0.98 (0.94–1.01)	0.218	0.94 (0.90–0.99)	0.027	0.94 (0.88–1.01)	0.95 (0.89–1.02)
Serum Potassium	0.93 (0.59–1.47)	0.764	1.07 (0.36–3.16)	0.907	0.20 (0.06–0.68)	1.79 (0.68–4.75)
eGFR	0.99 (0.98–1.00)	0.239	0.99 (0.98–1.00)	0.057	1.00 (0.98–1.02)	0.99 (0.98–1.00)
Low eGFR (<60 mL/min/1.73 m^2^)	1.18 (0.70–1.98)	0.540	1.46 (0.70–3.07)	0.317	0.37 (0.09–1.54)	2.11 (1.02–4.37)
Concomitant medication						
ASA	1.10 (0.66–1.82)	0.71	1.30 (0.61–2.79)	0.497	1.30 (0.27–6.39)	1.46 (0.63–3.43)
NSAIDs	3.17 (1.50–6.70)	0.003	1.49 (0.60–3.67)	0.387	0.79 (0.09–6.98)	1.72 (0.63–4.73)
Digoxin	1.86 (1.02–3.39)	0.042	1.70 (0.67–4.32)	0.262	3.70 (0.66–20.79)	1.00 (0.45–2.22)
Furosemide	3.40 (1.23–9.39)	0.018	6.33 (2.15–18.62)	0.001	6.35 (0.75–53.67)	4.02 (1.54–10.52)
Hydralazine	1.33 (0.57–3.10)	0.506	2.26 (0.87–5.87)	0.093	0.18 (0.02–1.80)	3.37 (1.24–9.15)
ISMN	1.18 (0.56–2.49)	0.665	1.86 (0.70–4.95)	0.213	-	2.91 (1.09–7.79)
Ivabradine	2.65 (1.31–5.38)	0.007	1.63 (0.69–3.82)	0.263	1.34 (0.23–7.89)	2.06 (0.92–4.59)
Trimetazidine	1.84 (0.57–5.90)	0.305	1.10 (0.27–4.42)	0.897	-	1.61 (0.37–7.05)
Medical history						
Hypertension	2.17 (1.27–3.70)	0.005	2.39 (1.10–5.17)	0.028	2.60 (0.51–13.30)	2.37 (0.94–5.97)
Ischemic heart disease	2.40 (1.18–4.90)	0.016	2.71 (1.03–7.12)	0.043	7.38 (1.6–33.82)	1.41 (0.55–3.63)
Atrial fibrillation	3.52 (1.87–6.66)	0.000	6.11 (3.37–11.08)	<0.001	18.90(5.51–64.76)	3.87 (1.56–9.59)
Diabetes mellitus	1.75 (0.93–3.30)	0.084	0.82 (0.38–1.79)	0.626	0.42 (0.07–2.39)	1.06 (0.45–2.50)
Chronic kidney disease	2.41 (1.04–5.62)	0.041	1.60 (0.60–4.29)	0.352	1.11 (0.12–9.95)	1.79 (0.58–5.48)

Exposed cohort: SGLT2i with low-dose conventional triple therapy group, reference cohort: high-dose conventional triple therapy. Abbreviations: SGLT2i, Sodium-Glucose Cotransporter 2 inhibitors; CSMBS, civil servant medical benefit scheme; NUH, Naresuan University Hospital; HF, Heart Failure; LVEF, Left ventricular ejection fraction; eGFR, estimated glomerular filtration rate; ASA, Aspirin at any dose; NSAIDs, Non-Steroidal Anti-Inflammatory Drugs; ISMN, isosorbide mononitrate.

**Table 4:** Multivariable Cox proportional hazards model of the clinical outcome.

Clinical Outcome	Cohort	Crude Event Rate (100 Patient-Years)	After IPTW Event Rate (100 Patient-Years)	Adjusted Hazard Ratio * After IPTW (95% CI)	*p*-Value
Composite outcome	expose	24.38	35.09	4.10 (2.07–8.13)	<0.001
	reference	4.99	6.52		
Dead	expose	6.09	8.03	6.40 (0.89–46.19)	0.065
	reference	0.89	2.46		
Hospitalization	expose	19.05	27.42	4.47 (2.20–9.06)	<0.001
	reference	5.17	4.97		

* Adjusted for age, BMI, study site, case type, NYHA functional class, systolic blood pressure, diastolic blood pressure, LVEF, eGFR, the administration of aspirin, NSAIDs, furosemide, hydralazine, isosorbide mononitrate, ivabradine, trimetazidine, and history of hypertension, ischemic heart disease, and atrial fibrillation.

## Data Availability

The data presented in this study are available on request from the corresponding author. The data are not publicly available due to privacy and ethical restrictions.
